# Managing supply chain uncertainty by building flexibility in container port capacity: a logistics triad perspective and the COVID-19 case

**DOI:** 10.1057/s41278-020-00168-1

**Published:** 2020-10-06

**Authors:** Dawn Russell, Kusumal Ruamsook, Violeta Roso

**Affiliations:** 1 University of North Florida, Jacksonville, FL USA; 2 The Pennsylvania State University, Pennsylvania, USA; 3 Technology Management and Economics Department, Chalmers University of Technology, 412 96, Gothenburg, Sweden

**Keywords:** Container freight transportation, Port logistics, Capacity, Uncertainty, Flexibility, Logistics triad, COVID-19

## Abstract

The contemporary supply chains in which container ports logistics operate are characterized by increased uncertainties driven by a range of factors such as socioeconomic factors and changing supply chain strategies in response to market dynamics. Recently, the occurrences and effects of these factors on global economic activities, and thus container port logistics, have been intensified by the COVID-19 pandemic. Enabling flexibility in port logistics is more important than ever to navigate uncertainties, now and in the post-COVID-19 world. This paper seeks to develop a conceptual framework to holistically capture different dimensions of container port logistics capacity. A systematic literature review method is employed to formulate a conceptual framework depicting the structure of various elements of capacity and the interplay among the logistics *triad* of transport carriers, port operators, and logistics service providers whose interactions and service capacities constitute the overall capacity of the system. The study reveals four dimensions of port logistics capacity, namely seaside interface, platform, landside interface, and system-wide, each of which consists of subelements that can be distinguished into static or adjustable. The proposed framework provides insights corresponding to the logistics triad roles and interactions within the system for understanding uncertainty characteristics, assessing various elements of capacity, and identifying potential levers to build flexibility into these interrelated capacity elements.

## Introduction

During the last two decades, the synergy between globalization and advanced information and communication technology has significantly changed the dynamics of the world’s marketplace. Companies, large and small, are no longer just importing and exporting products; they are also increasingly engaging in offshore outsourcing and constantly expanding in global markets (Coyle et al. 2017). Operating in this complex and globally dispersed business environment means greater dependence on a nimbler network of seaborne logistics systems that have by far been the most pervasive means of international freight movement. Containerized trade has been the fastest-growing segment of seaborne trade for many decades; since 1990, global container traffic has grown by an average of 10% annually (Fruth and Teuteberg 2017). The recent economic crisis has slowed it down but still in 2018 global containerized volumes increased by 4% to 146.4 million TEU (JOC 2020). Growth in containerized trade manifests itself in heightened demand for port services and growing operational challenges for ports, not only in physical handling of container traffic but also in providing quality services that correspond to today’s logistics and supply chain strategies (Behdani et al. 2020). One should also keep in mind that ports have an important social, economic, and environmental impact on their neighboring regions and generate added value and employment (Parola and Maugeri 2013; Charlampowicz 2019).

In parallel with the growth in containerized trade, the contemporary supply chains in which container ports operate are characterized by increased uncertainties. A range of factors contributes to such a phenomenon, ranging from socioeconomic factors (e.g., changing consumer demographics and rising trade protectionism) to changing supply chain strategies in response to market dynamics (e.g., near-shoring practices, supply base diversification, and adaptive transportation). While these factors played a part in the increased uncertainties long before the COVID-19 outbreak, their occurrences and effects on global economic activity, and thus container port logistics systems, have been intensified by the pandemic. In fact, industry experts describe today’s uncertainties to be “greater than at any time since the container revolution started in the late 1970s” (van Marle 2019).

In view of the supply chain uncertainties that will continue to be a common occurrence in the foreseeable future, it stands to reason that flexibility in seaport logistics capacity plays a vital role in mitigating the detrimental impacts of uncertainties now and in the post-COVID-19 world. A considerable amount of research has been undertaken on issues related to seaports’ capacity (e.g., Haralambides 2019; Parola and Maugeri 2013, Petering 2011; Theofanis and Boile 2009) as well as on uncertainties in supply chains and the importance of flexibility to combat such uncertainties, predominantly in a production context (e.g., van der Vorst and Beulens 2002; Swafford et al. 2006; Stevenson and Spring 2009; Kumar et al. 2006; Rodrigues et al. 2008; Skipper and Hanna 2009; Sanchez-Rodrigues et al. 2010; Singh et al. 2011; Fayezi et al. 2014; Vilko et al. 2014; Durach et al. 2015). However, studies relating these concepts in a service context, particularly in the increasingly important seaport logistics systems, are scarce. Relevant studies are also largely conducted from the perspective of a single logistics party, mainly port operators (e.g., Gharehgozli et al. 2016).

According to Altuntas Vural et al. (2019), triads^1^ can be very useful in understanding maritime logistics; the triads (in different arrangements) could contribute to the understanding of, e.g., value co-creation in maritime logistics. This makes sense because the concept of *maritime logistics* at seaports is by its very nature intermodal; i.e., containers that are unloaded into a seaport must be moved by other modes, primarily road and rail, to inland facilities. The formation of different arrangements in different maritime-related triads may provide important academic and practical insights.

Therefore, to fill the existing knowledge gap, this paper seeks to develop a conceptual framework to holistically capture different dimensions of container port logistics capacity. Developed on the basis of a systematic literature review, the framework considers a logistics triad consisting of carriers, port operators, and logistics service providers (LSPs), whose interactions and provision of their service capacities constitute the overall capacity of the system. Given the multimodal nature of moving goods into, through, and out of a seaport, port capacity is not created by one entity only; rather, it is created by the triad of transport carriers, port operators, and LSPs working in concert. Emphasizing the issues of capacity uncertainties in this triadic make-up of capacity, our framework delves into multidimensional capacity components embedded in a port logistics system under which the triad operates. The framework enables researchers and practitioners to understand the capacity components and thus identify uncertainty characteristics, and subsequently develop strategies to build flexibility to adjust capacity—a hallmark of competitive advantage in the container port logistics industry.

The rest of the paper presents the theoretical basis of supply chain uncertainty and flexibility, a research methodology narrative, the resulting conceptual framework with detailed explanation of capacity structure and elements, observations of uncertainties and flexibility strategies from literature, and finally conclusions and future research recommendations.

## Theoretical basis: supply chain uncertainty and flexibility

Our framework is founded on three main premises, informed by the concepts of supply chain uncertainty and supply chain flexibility. First, we consider flexibility as central in addressing capacity uncertainties in a container port environment. Second, understanding the structure of port capacity components is imperative to recognizing potential flexibility levers for managing capacity uncertainties. Third, flexibility in port logistics capacity is relevant not only to port operators, but also to transport carriers and LSPs that operate and interact with each other within the system. We highlight key premises of these concepts as follows.

### Supply chain uncertainty

Uncertainty is commonly portrayed in literature as a result of a combination of *exogenous turbulence* that is not within the control of an organization and *internal cognitive limitations* caused by absence of information, awareness, or clarity surrounding decision-making (Colicchia and Strozzi 2012; Durach et al. 2015; Emblemsvåg and Kjølstad 2006; Manuj and Mentzer 2008; Ritchie and Brindley 2007; Van der Vorst and Beulens 2002). *Risk*, on the other hand, while it is affected by exogenous turbulence like uncertainty, may or may not arise depending on the *likelihood of exposure* to the turbulence, *magnitude of impact*, and *uncertainty concerning the possible outcomes*. Thus, uncertainty is inherent in risk, or is among the preconditions for it to occur (Ford 2015; Rao and Goldsby 2009; Rodrigues et al. 2008; Sanchez-Rodrigues et al. 2010; Vilko et al. 2014).

In this study, we subscribe to these uncertainty and risk theories and define capacity uncertainty as a situation in which a decision-maker does not have sufficient information or clear perception of the nature of exogenous turbulences affecting port logistics capacity, of the possible solutions to capacity constraints, and/or of the possible outcomes after implementing different solutions.

### Supply chain flexibility

There is an overwhelming consensus in the extant literature that flexibility is the important capability to minimize the effects of external turbulence by allowing a system to quickly and cost-effectively respond to uncertainties in its environment (Bernardes and Hanna 2009; Engelhardt-Nowitzki 2012; Fayezi et al. 2014; Ming-Chang et al. 2014; Prater et al. 2001; Sheffi and Rice 2005; Singh et al. 2011; Skipper and Hanna 2009). Flexibility is consistently featured in extant literature as a key element of the related concepts of supply chain *agility* (Charles et al. 2010; Durach, et al. 2015; Fayezi et al. 2013; Gligor 2014; Kumar et al. 2006; Prater et al. 2001; Swafford et al. 2006), *resilience* (Jüttner and Maklan 2011; Kleindorfer and Saad 2005; Ponis and Doronis 2012; Sheffi 2005), and *responsiveness* (Prater et al. 2001; Thatte et al. 2013). Specific to the port context, we acknowledge Paixão and Marlow (2003), who apply lean production and agile theories in port operations. However, while they note different types of flexibility that port operators should be aware of to become agile, flexibility is not the primary focus of the study and the agile model focuses primarily on port operators. Based on these studies, agility in the context of this paper can be seen as the ability of the logistics triad to respond rapidly to external turbulence, while flexibility can be seen as a key element of agility that pertains to the logistics triad’s ability to adjust their assets and operations to different service types and volumes.

The multidimensional characteristics of flexibility are consistently discussed in extant literature, as reflected in the variety of supply chain flexibility taxonomies and frameworks proposed to articulate different *types* such as product, routing, and volume (Pujawan 2004; Singh et al. 2011); *elements*/*dimensions*, such as supply, market, logistics, operating systems, and organizational (Kumar et al. 2006; Sánchez and Pérez 2005; Singh et al. 2011; Yi et al. 2011); and/or *hierarchy* of flexibility, such as operational, tactical, strategic, and supply chain network (Engelhardt-Nowitzki 2012; Fayezi et al. 2014; Jin and Oriaku 2013; Stephenson and Spring 2007). Examples of types of flexibilities in a container port logistics environment are equipment capable of handling different types of cargoes, alternative modes of transport for hinterland access, diversified routes for ships to arrive at and depart from ports, and the number of ships and quantity of cargo that can be handled without disruption (Paixão and Marlow 2003).

Earlier research also puts forth *situational* characteristics of flexibility, whereby the applications of flexibility in a firm vary depending on the type of external turbulence, supply chain strategy, and intra- and interfirm capability available to the firm, all of which together dictate the trade-offs and synergies of flexible alternatives (Engelhardt-Nowitzki 2012; Fayezi et al. 2014; Kumar et al. 2006; Naim et al. 2006; Stevenson and Spring 2009; Swafford et al. 2006; Tachizawa and Thomsen 2007; Yi t al. 2011; Yu et al. 2012). It is observed that studies related to these concepts in a service context, particularly in the increasingly important seaport logistics systems, are scarce. Relevant studies are also largely conducted from the perspective of a single logistics party, chiefly port operators.

Drawing on the above precepts of flexibility, our framework is conceptualized to reflect the structure of a port logistics system under which the logistics triad of transport carriers (maritime and inland), port operators, and LSPs must make decisions amid increasing uncertainties. The focus is to use these theoretical bases as lens to guide decisions toward building capacity flexibility in the port logistics system.

## Systematic literature review methodology

The systematic literature review (SLR) methodology is employed to identify, select, and analyze relevant literature. SLR has been employed in organizational, managerial, and supply chain research, and is commonly carried out in multiple phases as conveyed by Denyer and Tranfield (2009), Rousseau et al. (2008), and Seuring and Gold (2012). Adapted from these studies, we conducted SLR in four phases namely: (1) review process planning, (2) literature search, including search terms, (3) literature selection and evaluation including, inclusion and exclusion criteria, and (4) data analysis and synthesis.

### Phase 1: review process planning

We aim to develop a conceptual framework to understand and analyze capacity in container port logistics. Then, through the lens of the framework, we present observations on characteristics of uncertainties and flexibility approaches employed by the port logistics triad. Given our objectives, a methodological framework is formulated to clarify the potential areas and themes to be investigated in conforming to the multidimensional and situational tenets of flexibility. The former includes *types* of flexibility (e.g., routing and ship fleet), *elements* (e.g., operating systems and handling equipment), and *hierarchy* (e.g., operational, tactical, strategic), while the latter includes type of external turbulence (e.g., weather, economic, and container freight demand condition) and operational capability available to the logistics triad (e.g., equipment technologies and labor skills). Accordingly, the questions we examined in the literature review are threefold: (1) what are the capacity components of a port logistics system? (2) what are the characteristics of capacity uncertainties in container port logistics? and (3) what are potential flexibility strategies for a port logistics triad, both individually and in consort?

### Phase 2: literature search

The purpose of the literature search is to create a comprehensive catalog of core contributions in relation to the review questions (Denyer and Tranfield 2009). The principal data sources were supply chain, logistics, and transportation academic journals in ABI/INFORM Collection and Academic Search Complete (Ebsco) databases, since these are global, well-known, and accessible databases of academic material. The search strategy was based on selected keywords that emerged through the scoping study by reviewing the literature. The combinations of these keywords, or search string, were constructed in four groups: (1) container, port, logistics, transportation, shipping, and maritime; (2) system, structure, process, and capacity; (3) uncertainty, risk, and congestion; and (4) flexibility, agility, adaptability, responsiveness, and resilience. The search combinations were applied within article content, title, abstract, and keywords on available full-text peer-reviewed documents.

### Phase 3: literature selection and evaluation

Only peer-reviewed articles published in the English language during the 15-year period 2005–2020 were selected for further evaluation, using the predefined inclusion and exclusion criteria summarized in Table 1. The admissibility of each article that met our inclusion criteria was confirmed, and the result was a total database of 56 peer-reviewed articles published in the said period.

**Table 1:** Data inclusion and exclusion criteria

Inclusion criteria	Exclusion criteria
Port logistics processes Container port operations Capacity and congestion issues in container freight transportation Container port competitiveness Logistics and transportation strategies in relation to uncertainties	Non-logistics and supply chain aspects of port logistics (e.g., engineering, macroeconomic, and public policy) Non-logistics capacity aspects of flexibility (e.g., variety of services, decision-making processes) Non-freight-oriented discussion (e.g., passenger cruises, information flows)

### Phase 4: data analysis and synthesis

The final set of articles was analyzed in depth following the *constant comparison approach* (Glaser and Strauss 1967; Strauss and Corbin 1998). Data were examined and coded^2^ on three levels, as described in Table 2, following Strauss and Corbin’s (1998) procedure of open, axial, and selective coding, respectively. The inductive process we employed has proven useful in situations such as the one studied here which focuses on understanding an emerging phenomenon, and/or on elaborating on existing ideas (Bryant 2002; Flint and Golicic 2009; Gouldin 2005; Hausman et al. 2010; Mello and Grawe 2009; Turner 1983). The constant comparative method is gaining support in business research, being employed in studies by Mallalieu and Palan (2006), Flint and Golicic (2009), Thomas and Esper (2010), and Hausman et al. (2010), among others.

**Table 2:** Three-level data coding description

Coding Level	Coding Focus and Output
Level 1	Focus: Dividing data into segments based on the commonalities that could reflect the roles and key processes of port logistics Output: A list of key container port logistics processes
Level 2	Focus: Making connections among the identified processes of moving a containerized freight through the port logistics systems. The focus is on exploring the conditions and interactions that influence these processes Output: A list of capacity requirements in terms of elements and their interactions in rendering services associated with the identified port logistics processes
Level 3	Focus: Selective coding of data using level 2 output as a guide to describe characteristics of capacity and associated uncertainties Output: A classification of capacity dimensions, and a descriptive condition of capacity uncertainties

## Capacity framework of container port logistics systems

**Fig. 1:**
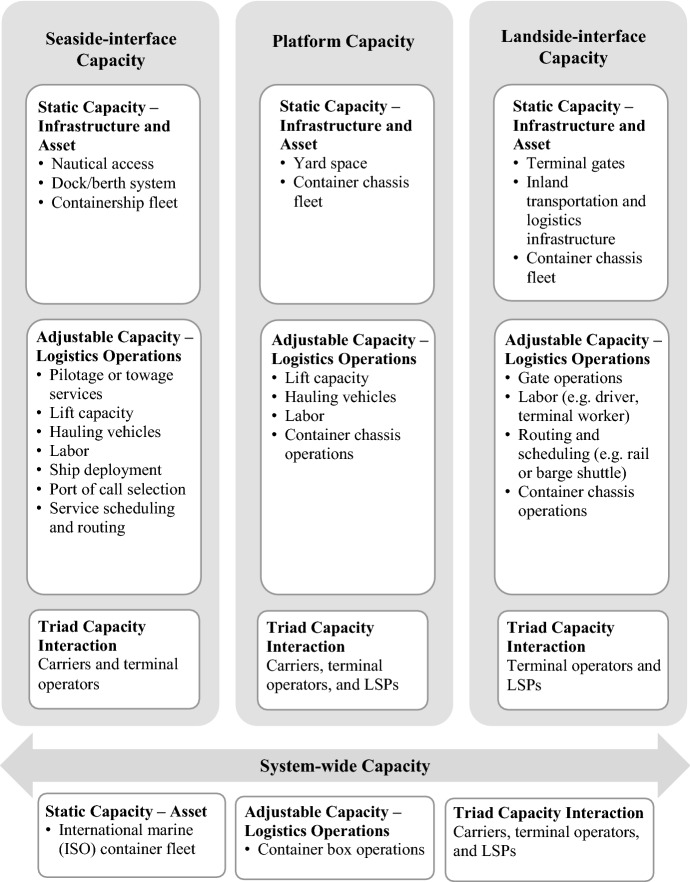
Capacity components of container port logistics systems

The framework shown in Fig. 1 depicts the capacity components in which uncertainty and flexibility are manifested. The findings reveal four dimensions of port logistics capacity as described in Fig. 1:

1. Seaside interface capacity, which deals with berthing ships at assigned berths and loading and unloading containers to and from vessels
2. Platform capacity, which deals with operations of terminal yards that provide capacity for container storage and other logistics services until they are loaded/offloaded onto/from ships
3. Landside interface capacity, which deals with freight movement between seaport facilities and inland logistics systems
4. System-wide capacity of international (ISO) maritime containers that flow through the entire port logistics systems

Each of the four capacity dimensions is conditioned by various subelements that can be either static or adjustable. The specific subelements of static and adjustable capacity for each of the four capacity dimensions (seaside interface, platform, landside interface, and system-wide) are noted in Fig. 1. The static–adjustable distinction is important because it affects the extent to which flexibility can be implemented to combat uncertainty. In general, flexibility in static capacity elements relies on *expansion* mechanisms to add buffer capacity (e.g., building a new container terminal or larger storage area), while flexibility in adjustable capacity elements relies on *adaptive* mechanisms to improve the utilization of existing capacity (e.g., scheduling of loading/unloading dock and handling equipment, reallocation of container equipment).

### Seaside-interface capacity dimension

Given the process of berthing ships at assigned berths and loading and unloading containers to and from vessels, this capacity dimension thus relies on the interaction between the maritime transport capacity provided by ocean carriers and the container terminal capacity provided by terminal operators. Vessel arrival delays cause disruptions to planned terminal operations. However, the impact of vessel arrival delay can be minimized by using an optimized schedule reliability model (Wang and Guo 2018) or through early detection of vessel delays (Sungil et al. 2017).

At the strategic level, ocean carriers provide maritime transport capacity via their containership fleet consisting of ships of varying sizes [e.g., ultralarge container vessels (ULCV), new Panamaxes, and specialized vessels (e.g., reefer containerships)]. The process of berthing these ships depends on the berth systems (e.g., number and configuration of berths), nautical access profile (e.g., tidal windows and water depths in access channel and port basins), and pilotage or towage services (e.g., availability of pilots and tug boats) (Bassan 2007; Layaa and Dullaert 2014; Notteboom 2006; Shuai et al. 2019; Tongzon 2007; Wiegmans et al. 2008; Yan et al. 2019).

The processes of loading and unloading containers to and from vessels depend on the terminal operators’ available capacity according to the expected workload for each incoming containership. The number of quay cranes (QCs) and manpower to be assigned is essentially determined by the minimum quayside productivity agreed upon in the contract between the ocean carrier and terminal operators (Ku et al. 2010; Legato et al. 2010; Monaco et al. 2009; Notteboom 2006).

### Platform capacity dimension

Given platform provisions of container storage and other logistics services, until containers are loaded/offloaded onto/from ships, the key to platform logistics processes is the capacity of hauling equipment that connects the seaside and stacking areas. Hauling equipment is required in transhipment processes, the import process (transporting containers from seaside to the stacking areas), and the export process (transporting containers from the stacking to seaside area). The types of hauling equipment used can be broadly classified into human-controlled e.g., trailer-trucks and straddle carriers (SCs)] and automated systems [e.g., automated lifting vehicles (ALVs) and automated guided vehicles (AGVs)] (Gharehgozli et al. 2016). In some cases where there is on-dock rail infrastructure, the containers are loaded directly onto railcars for transfer to near-dock operations (Pérez-Rodríguez and Holguín-Veras 2014).

The storage yard in terminals at most ports is divided according to the direction of containers passing through that terminal, namely transshipment, imports, and exports (Alessandri et al. 2009; Nishimura et al. 2009; Vis and van Anholt 2010). At some terminals, additional divisions are added for empty containers and special containers, such as dangerous goods, oversize containers, and reefers that need power supply. Specific parts of the yard may also be provided for a transloading station where containers are stuffed and stripped to consolidate/deconsolidate LCL containers. A segregation area, usually close to the inspection area and used for temporarily storing containers waiting to be inspected, is also provided in some container yards (Di Francesco et al. 2009; Longo 2010; Vis and van Anholt 2010).

Yard capacity is influenced by yard space and layout, ground crew manpower, and handling systems that depend on how containers are stored. Containers can be stacked on top of each other on the ground (*grounded operations*) or stored on a chassis (*wheeled operations*) (Dekker et al. 2013; Gharehgozli et al. 2016; Pérez-Rodríguez and Holguín-Veras 2014; Rodrigue and Booth 2013). Grounded operations require less area due to their higher storage density. However, they also involve more complex operations of handling systems (e.g., yard gantry crane) that stack and retrieve containers from their stacks, and hauling vehicles (e.g., automated guided vehicles, tow trucks with trailers, or straddle carriers) that move retrieved containers to barges, deep sea ships, trucks, or trains for further transportation (De Koster et al. 2009; Dekker et al. 2013; Gharehgozli et al. 2016; Lee et al. 2014; Meersman et al. 2012; Monaco et al. 2009). The benefits of the wheeled system are that containers stored on a chassis can be rapidly moved by terminal trucks. Since trucks can quickly charge or discharge chassis, the capacity of the terminal increases substantially (Gharehgozli et al. 2016). However, wheeled operations usually require a significantly larger fleet of chassis, and more land to store empty chassis and store containers on chassis (Dekker et al. 2013; Rodrigue and Booth 2013).

In the USA, container chassis are traditionally provided by ocean carriers. Containers are stored on chassis in a terminal yard until they are ready for pickup and delivery to ports and inland facilities. Thus, the ocean carrier absorbs the cost of supplying and maintaining chassis at the various locations serviced by the line. This model differs from that of most other countries, where the carrier, forwarder, or third-party logistics (3PLs) companies provide the chassis. The US model is changing, however, with an increasing share of chassis being owned by leasing companies (e.g., TRAC, Flexi-Van, and DCLI) instead of ocean carriers (FMC 2015; Rodrigue and Booth 2013).

### Landside-interface capacity dimension

Given the processes pertinent to freight movement between seaport facilities and inland logistics systems. This capacity dimension relies on the interaction between container terminal operators and inland logistics service providers. These may be arranged either by the carrier (carrier haulage by outsourced transporter or own subsidiary) or by shippers and forwarders (merchant haulage) (Frémont 2009; Van De Voorde and Vanelslander 2009).

Terminal operators provide land access to multiple modes of transportation for import and export containers, through several terminal gates linked to highways for trucks and trailers, or special platforms linked to railways (Van Asperen and Dekker 2013). Export containers typically arrive at the container terminal several days before the vessel arrives in port. Upon arrival, export containers go through an examination of required documents, container inspection, and security checks. Then, the location where the containers are to be stored in the container yard storage area is provided to the truckers, railroads, or barges. After dropping off the outbound export containers, sometimes logistics providers pick up the inbound import containers for transportation to inland destinations, something known as a *dual transaction* (Li et al. 2021 forthcoming; Lee and Kim 2010; Vis and van Anholt 2010). For import containers, the process is reversed, involving the transfer of import containers from the storage yard for hinterland transportation (Frémont 2009; Van De Voorde and Vanelslander 2009).

The capacity of a landside gate complex of a container terminal depends on the size and design of the complex as well as on gate operating systems. The former concerns the areas, layout, and number of service lanes, while the operating systems are related to the gate allocation and setting (e.g., fixed entry/exit or reversible), gate scheduling and appointment, and transaction processing (e.g., a one-stage approach, where all transactions are handled at a gate, or a two-stage approach where drivers complete a portion of the paperwork transactions electronically before arriving at a manned entrance gate to complete the entrance process) (Ozbas et al. 2014; Wang et al. 2018).

### System-wide capacity dimension

The capacity of international (ISO) maritime containers constitutes a system-wide capacity of port logistics systems. There are two main groups of owners—i.e., capacity providers—of containers, namely the ocean carriers (including global, niche, and feeder carriers) and container leasing companies. A small share of containers, usually old ones close to the end of their useful life, are owned by depot operators, who also handle, store, and repair empty containers. Some major shippers may also own or lease a relatively small fleet of containers for their dedicated use, although this is not very common (Theofanis and Boile 2009). The system-wide capacity of containers relies both on the fleet size and the utilization of containers. The latter is enhanced by reducing dwell times (e.g., in storage or in transit), dislocations, and damage (e.g., less maintenance), among other factors.

## Observations of capacity uncertainties and flexibility strategies

Viewed through the lens of the capacity framework described above and illustrated in Fig. 1, we can now observe capacity uncertainties that arise in the four capacity dimensions and systematically classify flexibility strategies employed in the corresponding dimensions by the port logistics triads.

### Seaside-interface capacity uncertainties and flexibility strategies

#### Capacity uncertainties

Uncertainties in port access can arise due to irregularities in pilotage or towage services and tide dependence that can create bottlenecks on the river or canal between the open sea and the port (Meersman et al. 2012; Notteboom 2006). Loading/unloading ships can also involve uncertainties with respect to estimated times of arrival of vessels, which prescribes the type and timing of capacity requirements, due to the low reliability of liner schedules. Examples of common reasons are inclement weather at sea, delays in the access to ports, congestion or labor strikes at the different ports of call, and domino effects of delays at previous ports of call (FMC 2015; Notteboom and Rodrigue 2008; Vernimmen et al. 2007).

#### Flexibility strategies

Faced with capacity uncertainties, container carriers may adopt strategic-level flexibility approaches of securing terminal capacity at key ports in their service schedules through long-term contracts with terminal operators, alliances to share terminal capacity among partners, and/or minority shareholdings or joint ventures in container terminals (Harrison and Fichtinger 2013; Notteboom 2006; Wiegmans et al. 2008). In this respect, shippers can minimize the uncertainties caused by schedule variability by contracting transport services with carriers that own (or partly own) the terminals they are visiting, are alliance members of terminals, or have long-term berthing contracts in place (Harrison and Fichtinger 2013).

Moreover, flexibility at operational and tactical levels can be achieved through adaptive vessel deployment and reshuffling of the order of ports of call on a certain loop. The former involves phasing in other vessels into the published schedule, while phasing out the delayed vessels to lay-by periods to be deployed again on demand (Notteboom 2006). The latter simply involves either a new string or new port calls along an existing string (Harrison and Fichtinger 2013; Vernimmen, Dullaert, and Engelen 2007).

Another infrastructure flexibility strategy conceived by the Korea Advanced Institute of Science and Technology (KAIST) (according to Shin and Lee 2013) is the use of new container handling systems called mobile harbors (MHs), a type of mobile floating port system equipped with a crane on board and other necessary facilities for off-shore container handling capability. The floating structures add capacity flexibility as they can be quickly deployed, removed, relocated, and expanded in many locations. The system also eliminates the need for a containership to directly call at a land-based berth (Kim and Morrison 2012; Shin and Lee 2013), reducing capacity restrictions related to nautical access (e.g., tidal windows and water depths) without undertaking time-consuming and resource-intensive infrastructure expansions (e.g., dredging access channels). Furthermore, according to Beuren et al. (2018), in Brazilian cases, new types of equipment (e.g., ship loaders) have allowed higher efficiency of cargo handling. Finally, advanced gate management systems, jointly optimized with related yard movements, reduce truck queues at port access (Li et al. 2021: forthcoming).

In terms of manpower, terminal operators seek capacity flexibility in their union agreements, such as the specification of gang sizes, work hours, workforce allocation, and operating practices. An additional, temporary workforce that is convened at short notice can also be used in peak periods (Monaco et al. 2009; Theofanis and Boile 2009).

### Platform capacity uncertainties and flexibilities

#### Capacity uncertainties

Uncertainties in yard capacity arise when there is a mismatch in container flows between quay and yard (e.g., due to low schedule integrity of vessels) or between yard and terminal gate (e.g., due to random arrivals of drayage truckers). Capacity uncertainties on the platform also arise from equipment maintenance and service failures, union labor strikes, chassis availability, carrier and dray service times, etc.

#### Flexibility strategies

Thus, platform capacity flexibilities depend on both yard operations (e.g., container stacking/retrieving processes, hauling equipment path flexibility and accessibility, and equipment availability) and yard integration with seaside and landside capacity. Terminal operators enhance hauling flexibility between yard and seaside through better coordination and real-time control of hauling equipment. The digitalization in the maritime logistics sector, such as automation, big data and tools for vehicle tracking and tracing, transponders, laser scanners, or GPS in combination with RFID, offer numerous opportunities. Smart container technologies and real-time tracking of cargo can increase transparency along the transport route from sender to recipient (Fruth and Teuteberg 2017). Terminal operators are able to use a smaller hauling equipment fleet, reduce (empty) travel times, and better manage the inherent operational variability in data-driven scheduling (Gharehgozli et al. 2016).

Another example of a *flexibility strategy* implemented at container ports focuses on chassis capacity, using a pooling approach that requires all chassis on-site to be part of a port-wide gray pool. This strategy helps eliminate unproductive chassis operations (e.g., truck trips, wait times, and yard storage space) that arise because different chassis pools do not necessarily serve all the terminals in the port complexes. The Ports of Los Angeles/Long Beach and New York/New Jersey, for example, have set up a “pool of pools” that allows all chassis in a port complex to be gray but with multiple pool managers (e.g., leasing companies and ocean carriers). This permits chassis in different pools to be used interchangeably through a chassis use agreement (FMC 2015).

### Landside-interface capacity uncertainties and flexibilities

#### Capacity uncertainties

Uncertainties usually arise when there is limited gate capacity, time-varying demand (e.g., low traffic during nights and weekends), and unavailability of chassis (e.g., due to dislocation or damage) that could lead to congestion not only at the gate but also on streets outside port terminals (Dekker et al. 2013; Guan and Liu 2009; Meersman et al. 2012).

#### Flexibility strategies

Strategically, one way of improving landside-interface capacity is by extending the terminal gate into the hinterland through the addition of inland terminals with flexible rail services (Hu et al. 2019), or dry ports that are directly connected to the seaport terminals with a high-capacity transport mode such as intermodal rail (Khaslavskaya and Roso, 2020). In such a system, customers can instead drop or pick up their containers at the inland terminals. Dry ports can also provide services such as customs, security inspections, pre-assembly, labeling, and packaging (Khaslavskaya and Roso 2020; Roso et al. 2015; Bask et al. 2014).

In terms of gate capacity, flexibility can also be improved by increasing the number of gates. However, this approach is subject to land availability and yard-handling capacity (Guan and Liu 2009; Mazouz et al. 2017). Operationally, gate capacity flexibility can be achieved by improving gate utilization through appointment systems, allowing terminal operators to spread the expected work volume more evenly throughout the day and plan resources accordingly (FMC 2015; Gharehgozli et al. 2016; Guan and Liu 2009; Ramírez-Nafarrate et al. 2017). However, from an inland carrier’s perspective, an appointment system, particularly in traditional systems that often must be scheduled days in advance, diminishes flexibility as truckers are restricted to the times they can enter the port (FMC 2015; Islam and Olsen 2014). Dynamic appointment systems, such as the Freight Advanced Traveler Information System (FRATIS) used in the Los Angeles/Gateway Cities region in Southern California, could remedy this flexibility conflict (FMC 2015). Zhang et al. (2016) advocate the value of information and ICT systems that can reduce lead times and transportation costs, and increase reliability. These authors refer in particular to the importance of information sharing between the vessel prior to arrival and the transportation planners at the hinterland side.

Regarding the container chassis needed for inland movements, terminal operators are experimenting with the concept of the *chassis exchange terminal* (CET) (Dekker et al. 2013). A CET is an off-dock terminal where truckers deliver a chassis with an export or empty container and collect a chassis with an import or loaded container. The truckers shuttle the chassis of a pool between the CET and seaport terminals, allowing the consolidation of containers with the same destination to be dropped off and picked up. This idea could improve capacity flexibility since truck carriers can share the available capacity of both trucks and chassis pool, and do not rely on terminal personnel or equipment capacity for chassis exchange operations (Dekker et al. 2013).

For ocean carriers, flexibility in chassis capacity is achieved by participating in various types of chassis pooling arrangements in which two or more carriers contribute and agree to share chassis when moving their containers to and from intermodal facilities in a specific location or region (FMC 2015; Rodrigue and Booth 2013).

### System-wide capacity uncertainties and flexibilities

#### Capacity uncertainties

The number of empty containers available in a port is uncertain because ocean carriers do not know precisely when their customers will return the containers and how much time will be needed to move them to ports (Di Francesco et al. 2009). Uncertainties are further accentuated by the directional imbalance of container trades between export-oriented and import-oriented regions. This is notably acute in the Transpacific and Asia–Europe–Asia trade lanes. As a result, container availability is unbalanced, with an accumulation of empty containers in import-oriented areas and shortages in export-oriented areas (Di Francesco et al. 2009; Pérez-Rodríguez and Holguín-Veras 2014; Theofanis and Boile 2009).

#### Flexibility strategies

Ocean carriers add flexibility to container availability at various management levels, mainly through expanding container fleet size, repositioning of empty containers from surplus to deficit regions, and leasing containers (Theofanis and Boile 2009).

Repositioning of empty containers could improve container utilization, thus equivalently increasing fleet capacity. Ocean carriers adopt advance planning approaches and engage in better coordination regarding the use of empty containers, which enables them to borrow a container from a partner company and avoid hauling one from a remote location. These approaches have greatly benefited from Internet-based bulletin boards that help expedite the process of identifying and exchanging containers (Pérez-Rodríguez and Holguín-Veras 2014).

Because overseas repositioning of empty containers is normally performed by scheduled containerships mainly carrying profit-generating loaded containers, this flexibility approach is subject to residual vessel spaces that are not occupied by loaded containers (Di Francesco et al. 2009; Dong and Song 2009). In addition, since several terminals operate satellite empty container depots to gain additional storage capacity and avoid congestion at the gates, this flexibility approach is also influenced by the inland transport capacity for movements between marine terminals and satellite storage depots, as well as movements between different storage depots (Theofanis and Boile 2009).

Instead of owning containers, ocean carriers can add capacity flexibility through leasing arrangements. The other triad members, notably inland transport carriers and shippers (Altuntas Vural et al. 2019), can also enhance container capacity flexibility in this way, instead of or in addition to relying on containers provided by ocean carriers. Arrangements can take the form of long-term leases (also called dry leases) for extended use of containers without management service by the lessor; medium-term master leases (also called full-service leases), where the leasing company is responsible for the full management of the fleet (repositioning, maintenance, and repair); and short-term leases (also called spot market leases) that do not involve any management services by the lessor (Theofanis and Boile 2009).

## Conclusions and future research recommendations

Port logistics system capacity is a multifaceted problem requiring multifaceted solutions. This study offers a framework for understanding capacity elements in container port logistics systems. The four dimensions of the port logistics capacity framework systematically portray this multifaceted problem in a way that allows port logistics triads, namely transport carriers, port operators, and LSPs, to identify potential bases for flexibility strategies congruent with their own contextual conditions. The container port logistics industry is still young, and there remain many improvements to be pursued—capacity flexibility being amongst them. Capacity flexibility combats port congestion and keeps freight moving. This is increasingly important as we navigate the uncertain waters of the COVID-19 pandemic. Ports are necessarily evolving their operations and protocols, and the need for flexibility to combat uncertainty is at an all-time high. This work contributes to that mission.

A key insight regarding capacity flexibility is that there are static and adjustable components of container port capacity, and each offers opportunity to better manage and build capacity. Flexibility in static components relies on expansion mechanisms, while flexibility in adjustable components relies on adaptive mechanisms to improve the utilization of existing capacity. Further, this work uses a theoretical lens of uncertainty that illuminates the concept of “exogenous turbulence,” which refers to the uncertainty elements that are not under the control of the organization. This is particularly applicable to the port industry, as evidenced by the fact that a triad of organizations make up the port system. The other element of uncertainty that plays heavily is “internal cognitive limitations,” referring to the lack of information, awareness, and clarity surrounding decision-making. This is an ongoing challenge in the triad of capacity management in container port logistics.

This work can be extended in several significant areas. One area is the extension of this introduction to modeling port capacity as a triad which consists of multiple companies, each of which plays a key role in the ability of a port to service its community. Such modeling should be enhanced by studying empirical data. Further, the impact of capacity flexibility on port throughput should be considered. Case study assessments from the perspective of the triad could provide insight into the decision factors that impact overall port logistics capacity and demand for port services.

More research is also needed to determine how total system capacity can become more flexible. It is believed that port capacity is not just the sum of resources from landside, seaside, and platform, but the concept rather involves more complicated interactions of the various components. Furthermore, capacity flexibility is expected to vary among different ports, countries, continents, and freight types.

Given the land-locked status of many port authorities and the limited resources to expand port assets in many countries, researchers should explore ways to increase port capacity flexibility by other, less conventional means. These means may include training, leadership and motivation, strategic port positioning, hinterland dry ports, information systems, and other means that repurpose existing resources toward expanded capacity.

## Footnotes

1 Triad as a concept was coined in 1989, and is defined as the smallest unit of a business network; it is a useful tool to study relationships within larger business networks (Altuntas Vural et al. 2019).

2 a process of conceptual abstraction by assigning concepts (codes) to the observed data and phenomenon during data analysis.
